# Serum testosterone levels of HbSS (sickle cell disease) male subjects in Lagos, Nigeria

**DOI:** 10.1186/1756-0500-4-298

**Published:** 2011-08-17

**Authors:** Emmanuel K Abudu, Sulaiman A Akanmu, Oyetunji O Soriyan, Akinsegun A Akinbami, Adewumi Adediran, Titilope A Adeyemo, Charles C Okany

**Affiliations:** 1 Department of Morbid Anatomy & Histopathology, Olabisi Onabanjo University, Obafemi Awolowo College of Health Sciences, Sagamu, Ogun State, Nigeria; 2 Department of Haematology and Blood Transfusion, College of Medicine, University of Lagos, Nigeria; 3 Department of Chemical Pathology & Immunology, College of Medicine, University of Lagos, Nigeria; 4 Department of Haematology and Blood Transfusion, Lagos State University, College of Medicine, Ikeja, Nigeria

## Abstract

**Background:**

Infertility is a major problem in sickle cell disease patients, especially in males. In addition to low serum testosterone, other abnormalities involving the accessory sex organs, such as the seminal vesicles and the prostate gland, as well as marked decrease in ejaculate volume may be observed in male HbSS patients. Hence, the need to study the role of sex hormones as a cause of infertility in male HbSS patients.

**Methods:**

An unmatched case-control study was performed using seventy-five consenting subjects from Lagos University Teaching Hospital. These included 47 patients with haemoglobin phenotype SS from the Sickle cell clinic and 28 volunteered medical students and members of staff with haemoglobin phenotype AA. Demographic data were obtained using a self-administered questionnaire. A total of 5 mls of blood was collected from each subject between 9.00 am & 11.am, and assayed for serum testosterone concentration.

**Results:**

The concentrations of serum testosterone in HbSS patients ranged from 0.2 to 4.3 ng/ml with a mean of 1.28 ± 0.72 ng/ml whilst the values in HbAA controls ranged from 1.2 to 6.9 ng/ml with a mean of 2.63 ± 1.04 ng/ml. Seven (25.0%) of the 28 controls had serum testosterone concentration lower than the quoted reference (normal) range whereas 44 (93.6%) of the 47 HbSS subjects had serum testosterone concentration lower than the reference range.

**Conclusion:**

Overall, subjects with HbSS have significantly lower mean serum testosterone than HbAA controls.

## Background

Homozygous sickle cell disease is a genetic disorder caused by a point mutation in the beta globin gene, which results in the substitution of valine for glutamine [1]. The resultant haemoglobin variant, HbS, polymerizes at low oxygen tension, causing the characteristic sickle deformity of the red cells, the main aetiopathogenetic feature of this disease [2].

### Infertility in Sickle cell disease

Infertility is a known complication in males with sickle cell disease. This has been attributed to relative primary gonadal failure, impotence, and priapism, delayed or impaired sexual development [3]. The ejaculate volume, sperm motility, sperm density, and normal sperm morphology were significantly reduced in the patients when compared with the control subjects [4]. Most importantly, primary testicular failure is characterized by low levels of testosterone in which infertility results mainly from diseases or conditions that primarily affect and destroy the testis [5]. The low levels of testosterone results in reduction of fertility which is aggravated by impotence, secondary to earlier priapism [6].

Oligospermia due to local ischaemia caused by sickling phenomenon and tissue hypoxia have been reported in sickle cell disease patients [7]. Hypopituitarism reported in HbSS disease, results from intravascular thrombosis and pituitary infarction [8]. Sickle cell disease is associated with high folate demand. This with zinc deficiencies have been implicated in pituitary and primary gonadal dysfunction [9].

It is noted that reduction in semen volume, sperm count and motility in sickle cell male patients are probably attributed to low level of testosterone [10].

The aim was to determine the testosterone levels in sickle cell disease male patients compared with levels in HbAA controls, using enzyme linked immunosorbent assay (ELISA).

## Methods

This was an unmatched case-control study using homozygous sickle cell disease male patients attending the sickle cell clinic of the Lagos University Teaching Hospital and HbAA controls consisting of apparently healthy medical students and members of staff from the same health institution. Seventy-five subjects between 15 - 45 years age groups, consisting of forty seven (47) male patients with homozygous sickle cell disease (HbSS) and twenty-eight (28) volunteered male controls with HbAA phenotype were recruited consecutively between April 2008 to August 2008. Approval was obtained from the institution's research and ethics committee. Consenting participants were asked and aided to fill the structured questionnaires, consisting of demography information and history of smoking, consumption of alcohol, drug use apart from the usual routine medications for sickle cell disease patients and medical problems such as hypertension, diabetes mellitus and rheumatoid arthritis.

### Exclusion criteria

1) Patients with homozygous sickle cell disease not in steady state .2) Subjects on special drugs such as chemotherapy, anabolic steroids, cimetidine, spironolactone, phenytoin, sulfasalazine and nitrofurantoin that could affect serum testosterone assay.3) Subjects with history of recent or chronic consumption of alcohol and smoking of cigarette.4) Healthy controls with obvious medical problems such as diabetes mellitus, hypertension, rheumatoid arthritis,5) Grossly haemolyzed, lipaemic, icteric or improperly stored serum.

### Inclusion criteria

1) HbSS patients in steady state.2) Apparently healthy HbAA control groups with no obvious medical problems such as diabetes mellitus, hypertension, rheumatoid arthritis

### Collection of sample

A total of 5 mls of blood sample was collected in an unanticoagulated bottle from Hb SS subjects during period of steady states and the Hb AA controls. All samples were collected between 9.00 and 11.00 am due to the circadian rhythm of testosterone. The sera were separated and stored at -20°c on the same day of collection until ready to be analyzed for testosterone.

### Analysis of Data

The descriptive data were given as means ± standard deviation (SD). Distributions of serum testosterone concentrations in cases and controls were compared using independent sample t-test. The differences were considered statistically significant when p value obtained was < 0.05.

## Results

A total of 75 subjects consisting of two groups, 47 HbSS and 28 HbAA (controls) were recruited for this study. The age range was 17 to 41 years. Most HbSS subjects were in the 20-24 years age group (51.1%) with a mean age of 24.25 ± 5.27 years while most of the controls were in the age group 30-34 years (39.3%) with a mean age of 29.5 ± 2.12 years (p = 5.16 × 10^-5^) [Table 1].

**Table 1:** Age and Serum Testosterone Concentrations in HbSS and HbAA Subjects

AGE(Years)	HBSS SUBJECTS			HBAA SUBJECTS			All subjects
	n	LOW*	NORMAL**	n	LOW*	NORMAL **	
15-19	12	12	-	-	-	-	12
20-24	24	23	1	7	2	5	31
25-29	5	3	2	8	2	6	13
30-34	4	4	-	11	3	8	15
35-39	1	1	-	1	-	1	2
40 and above	1	1	-	1	-	1	2

**TOTAL**	**47**	**44 (93.6%)**	**3(6.4%)**	**28**	**7 (25.0%)**	**21(75.0%)**	**75(100%)**

Reference Concentrations of Serum Testosterone = 2 to 10 ng/ml

*Low serum testosterone level ≤ 2 ng/dl

**Normal serum testosterone level > 2 ng/dl.

The concentrations of serum testosterone for homozygous sickle cell disease patients ranged from 0.2 to 4.3 ng/ml whereas the concentrations of serum testosterone for HbAA patients ranged from 1.2 to 6.9 ng/ml. Most of the HbSS patients, 44 out of 47 (93.6%) had serum testosterone concentrations below the normal range when compared with HbAA of 7 out of 28 (25.0%) [Table 2]. The mean serum testosterone concentration for HbAA controls of 2.63 ± 1.04 ng/ml was significantly higher than mean of 1.28 ± 0.72 ng/ml obtained for HbSS patients (p = 2.8 × 10^-7^) [Table 2]. The reference range provided by the manufacturer (BIOCHECK, INC) was 2 to 10 ng/ml.

**Table 2:** Age and Mean Serum Testosterone Concentrations of HbSS and HbAA Subjects

Age Groups		HbSS		HbAA
(Years)	Numbers of subjects	Mean Serum ± SD Testosterone(ng/ml)	Numbers of subjects	Mean Serum ± SD Testosterone(ng/ml)
**17-22**	23	1.139 ± 0.575	3	2.833 ± 0.551
**22-28**	13	1.638 **± **0.973	8	2.228 ± 0.565
**29-above**	11	1.073 ± 0.514	17	2.753 ± 1.257

**Total Average**	**47**	**1.283 ± 0.514**	**28**	**2.625 ± 0.791**

There was no statistically significant difference in the serum testosterone level when the data were stratified by age-group (17-22, 23-28 and 29-above) (Table 1)

However, subjects less than 22 years old and older than 29 years old with normal haemoglobin phenotype had significantly higher mean serum testosterone concentration than those homozygous sickle cell disease patients of the same age groups [*p *= 0.000 < 0.05.] Table 2

The mean age of puberty for sickle cell anaemic (HbSS phenotype) patients was 17.65 ± 1.86 years which was significantly higher than the mean age of puberty for the controls (HbAA phenotype), 14.1 ± 0.93 years (p = 5.6 × 10^-13^).

Twenty-five (53.2%) HbSS subjects had history of priapism; most commonly seen in the ages between 10 and 14 years while the remaining 22 (46.8%) had no history of priapism.

## Discussion

The result of this study showed that serum testosterone levels are significantly reduced in sickle cell male patients compared to matched controls. This finding is similar to work done by Dada et al [8], Abbasi et al [10] and Parshad et al.[11] The low levels of serum testosterone may be a reflection of hypogonadism secondary to hypopituitarism [12]. Hypopituitarism in HbSS disease may result from intravascular thrombosis and pituitary infarction [12].

Other contributing factors like repeated testicular infarction which may be total or segmental has a probable underlying mechanism of testicular failure, impaired fertility, and reduced serum testosterone in patients with sickle cell disease [12,13]. The low testosterone levels may manifest as decreased muscle mass and bone mineral density, increased fat mass, central obesity, insulin resistance, decreased libido and energy, irritability, and dysphoria [13].

Most of the HbSS subjects were relatively younger with mean age of 24.2 years compared with HbAA controls (29.5 years). Although, cases and control groups differ very significantly by age, the mean serum testosterone concentration in controls for those below 22 years old and above 29 years old was significantly higher than homozygous sickle cell disease patients of the same age groups. From the foregoing, the difference may be explained by small sample size of the control subjects relative to the cases group. Moreover, this difference in age with relationship to the serum testosterone concentration may be reduced slightly because testosterone levels tend to fall with age with the decline beginning at about age 30 [14,15].

Most of the HbSS subjects studied had late onset of puberty with a mean age of 17.65 years compared with HbAA controls (14.1 years) similar to findings reported by Consuelo et al [16].

Twenty-five (53.2%) of the HbSS patients had history of priapism with ages between 10 and 14 years most commonly affected. This finding concurs with other studies done by Ibidapo et al in Lagos, Nigeria, Farrukh Sheh and Renard Davies in London, UK who recorded relatively high values ranging from 40% to 67.0% cases of priapism in sickle cell anaemic patients [17,18].

Agbaraji et al and Osegbe D.N. et al reported that the fertility indices such as ejaculate volume, sperm motility, sperm density, and normal sperm morphology were significantly impaired in sickle cell anaemic patients when compared with the controls, which further corroborated the role of reduced serum testosterone concentration in the investigation of infertility cases [4,19].

Some of the limitations of our study are, appreciable numbers (25.0%) of matched HbAA controls had serum testosterone levels below the manufacturer's quoted reference range and the facts that samples could not be collected for serum testosterone levels in all the HbSS and HbAA subjects at the same time. This is of importance because testosterone concentrations falls with age with the decline beginning at about age 30 and is often progressive, leading to levels in the 60 s-80 s that are below the reference range for young adults [13].

Other reasons for the relatively low serum testosterone levels in those affected controls may be attributed to poor cold chain storage system from the country of manufacture to Nigeria. Improper storage especially in conditions of erratic power supply which is found in the developing country like Nigeria, diurnal rhythm of testosterone secretions, environmental influences and taking of androgen suppressing drugs [20]. Although, the effects of these factors were minimal in our study as evidenced by validation tests done using sets of controls provided by the manufacturer. In addition, supposedly healthy controls may be suffering from diseases such as diabetes mellitus, rheumatoid arthritis and thyrotoxicosis which may secondarily lead to reduced testosterone from hypogonadism.

The day to day variation in the measurement of serum testosterone levels encountered in our study could be avoided as much as possible by recruiting patients with homozygous sickle cell disease at sickle cell clinics with high turnover of patients and probably conducting the assay within a short possible time. The marked diurnal rhythm in total testosterone should also be taken into consideration as widely reported [2,20]. As testosterone concentration may fluctuate markedly both seasonally and from day to day, it may be judicious to measure levels on more than one occasion [20]. Importantly, all testosterone concentrations should be measured at least twice to make the diagnosis of hypogonadism [21].

Another factor that may contribute to assay of serum testosterone is variation in types of kit used and thus, it is important to compare and validate efficiency and potency of other kits for future detection of serum testosterone in sickle cell anaemia in Nigeria [21].

The effect of varying climate change could not be ignored as a possible slightly reducing effect of a tropical environment on the circulating total testosterone levels in Zambian African males compared to men of the temperate regions has been reported [22]. In addition, our study is limited by the inability to totally relying on information given by patients concerning abstinence from certain drugs, cigarette smoking and alcoholism that may affect the testosterone assay. It has been reported that medications, including those that affect spermatogenesis such as chemotherapy, anabolic steroids, cimetidine, spironolactone; those that decrease FSH levels such as phenytoin; and those that decrease sperm motility such as sulfasalazine and nitrofurantoin should be avoided prior to serum testosterone assay to ensure accurate and precise results [20].

The frequent inaccuracies in testosterone measurements reported widely demand standardization of testosterone assay with the aim of defining performance criteria that cover the full range of expected values, from children to adult males and females; define reference intervals for testosterone in adults and children of both sexes; and developing guidelines and protocols to ensure uniform patient preparation and handling of samples before they are assayed. It is our hope that future studies in this environment will eliminate the mentioned limitations and will involve age, and sex matched homozygous sickle cell diseases patients and control subjects with a larger number of subjects. Other assay methods such as radioimmunoassay (RIA), competitive protein binding methods, established double isotope derivative methods and chromatography mass spectrometry should be encouraged to reveal other undiscovered findings about serum testosterone assay in patients with sickle cell disease as well as confirming findings of earlier study.

## Conclusion

Overall, subjects with HbSS have statistically significantly lower mean serum testosterone than HbAA controls. HbSS men are significantly more likely to have serum testosterone below the normal threshold, resulting in delayed onset of puberty and impairment of sexual development observed in HbSS cases.

## Limitations

Reliability on the puberty age and history of priapism provided by the subjects. Effects of climatic change on subjects and storage of the testosterone kit could not be totally ignored. Total reliability on the claim of not being on any medications affecting serum testosterone assay such as chemotherapy, anabolic steroids, cimetidine, spironolactone, phenytoin, sulfasalazine and nitrofurantoin as well as abstinence from cigarette smoking and chronic alcohol consumption could not be ascertained.

## Conflict of interest

The authors declare that they have no competing interest, study was self-funded.

## Authors' contributions

All authors read and approved the final manuscript. AA - Conceived the topic, EK - Did Literature search, AA - Collected the sample, SO - Carried out the assay, AT - entered the data, AA - data analysis, OC - final review of the manuscript
